# Factors associated with minimal meal frequency and dietary diversity practices among infants and young children in the predominantly agrarian society of Bale zone, Southeast Ethiopia: a community based cross sectional study

**DOI:** 10.1186/s13690-017-0216-6

**Published:** 2017-11-13

**Authors:** Mekonnen Tegegne, Semere Sileshi, Tomas Benti, Mulusew Teshome, Haile Woldie

**Affiliations:** 1 Department of Nursing, Madda Walabu University, Bale Goba, Ethiopia; 2 Department of Public Health, Madda Walabu University, Bale Goba, Ethiopia; 3 Department of Human Nutrition, Institute of Public Health, College of Medicine and Health Sciences, University of Gondar, Gondar, Ethiopia

**Keywords:** Aged 6–23 months, Meal frequency, Dietary diversity, Associated factors

## Abstract

**Background:**

Poor infant and young child feeding (IYCF) practices in the first 2 years of age are among major causes of childhood malnutrition, in developing countries including Ethiopia. It results in irreversible outcomes of stunting, poor cognitive development, and significantly increases risks of many chronic and infectious diseases. This study was intended to assess factors associated with minimum meal frequency and minimum dietary diversity practice among children aged 6–23 months in the predominantly agrarian society of Bale zone, Southeast Ethiopia.

**Methods:**

A community based cross sectional study was employed from January to June 2016. An interviewer administered, pretested and structured questionnaire was used to collect data. Multi-stage sampling followed by a systematic random sampling technique was used to include study subjects. Data was entered using Epi info version 3.5.3 and analyzed by SPSS version 20. In the logistic regression, both bivariate and multivariate analyses were carried out to identify factors associated with minimum meal frequency and minimum dietary diversity scores. All variables with *P*-values of <0.2 in the bivariate were earmarked for the multivariate analysis. Both Crude Odds Ratio (COR) and Adjusted Odds Ratio (AOR) were computed at 95% Confidence Interval (CI) to determine the strength of associations. In the multivariate analysis, variables at P–Values of <0.05 were considered as statistically significant with minimum meal frequency and dietary diversity practice.

**Result:**

A total of 801 infants and young children aged 6–23 months and their mothers participated in the study. The overall prevalence of minimum meal frequency and minimum dietary diversity practice was 68.4% [95% CI: 0.652, 0.716] and 28.5% [95% CI: 0.254, 0.316], respectively. Child age (AOR = 0.29; 95% CI: 0.28, 0.94) and parity of mother (AOR = 2.8; 95% CI: 1.11, 7.50) were independently associated with minimal meal frequency. On the other hand, mothers educational level (AOR = 0.52; 95% CI: 0.28, 0.94), child illness in the past 1 week (AOR = 0.44; 95% CI: 0.26, 0.73) and maternal counselling on IYCF practice during postnatal care (PNC) visits (AOR = 2.6; 95% CI: 1.59, 4.45) were factors statistically associated with dietary diversity practice in the study area.

**Conclusion and recommendations:**

Compliance to recommended minimum meal frequency and diversified diets was low in this study community. Minimum meal frequency was associated with the age of child and parity of mother. But, mothers’ education, child illness in the past 1 week, and maternal counseling on IYCF during PNC visits were factors associated with minimum dietary diversity practice. Improving the level of maternal and child health care utilization, increasing the educational level of mothers and providing health and nutrition counseling on IYCF during maternal PNC service visits are vital interventions to improve IYCF practices in the predominantly agrarian society of Bale zone, Southeast Ethiopia.

## Background

Appropriate feeding practices in the first 2 years of age are important measures to meet nutritional requirements [1] and improve physical growth and cognitive development [2]. The most recent preliminary results showed that improper IYCF practices in this windows period are among the major causes of childhood malnutrition [3] resulting in permanent outcomes of stunting, poor cognitive development, and significantly increases risks of many chronic and infectious diseases [4–6]. The potential negative impact of malnutrition during this critical period is not limited to childhood life [7] rather it diminishes individual level of productivity during adulthood [8], negatively affecting the future social and economic development of countries [9] and leading the vicious cycle of intergenerational malnutrition [10].

Globally, childhood malnutrition is the most pressing public health problem [11], predominantly in developing countries as it has primarily been directly or indirectly responsible for 60.0% of the 10.9 million annual child deaths [12]. More than two–thirds of these deaths are associated with inappropriate child feeding practices which occur during the first 2 years of life [13]. Furthermore, malnourished children are at higher risks of repeating grades in school and dropping out from schools which is costly to the families and educational systems of countries [14]. In Ethiopia, malnutrition is a significant public health problem among infants and young children. According to the 2011 demographic and health survey (DHS) report of the country, about 44.4, 28.7 and 9.7% of under 5 years of age Ethiopian children were stunted, underwieght, and wasted, respectively [15].

The quality of a child’s food is dependent on meal frequency and food groups contained in the diet. However in most of developing countries, infants and young children are directly introduced to regular household diets made of cereal or starchy foods [16] which are poor in quality. Moreover, the 2014 report of the International Food Policy and Research Institute on global nutrition situation indicated that inorder to attain nutritional requirment and prvent deficiencies, child feeding practie in the first 2 years of age needs special attention in Africa [13]. In the Sub–Sahara African region, inadequate IYCF practices are among causes of high childhood morbidity and mortality [17].

The Government of Ethiopia has been implementing a number of strategies [the 2004 National Strategy for IYCF, the 2005/2006 National Nutrition Strategy, the 2008 National Nutrition Program) to improve the levels of IYCF practices [18–20]. However, the country is among nations well known for their low levels of meal frequency and poor dietary diversity practices. According to research reports in Ethiopia, the magnitude of minimum meal frequency and minimum dietary diversity practices among children aged 6–23 months ranges from 44.7–82.0% and from 10.8–39.1% [15, 21, 22].

The cause of inappropriate IYCF practices is multi–factorial and has diverse contributing factors [5]. Previous studies that aimed at revealing the determinants of inappropriate IYCF practices among infants and young children reported that socio-economic and demographic characteristics [age and sex of the child, residence, mothers level of education and occupation, occupational and educational status fathers, household family size, household wealth index]; cultural and traditional beliefs related factors [traditions, cultural beliefs, maternal perception IYCF, taboos on IYCF]; co–morbidity and health care utilization characteristics of children and mothers’ [child illness in the past 1 week, child growth monitoring participation, antenatal (ANC) and mothers’ counseling on IYCF during PNC service visits] [23–32] were factors influencing IYCF practices. Thus, all these factors impacted minimum meal frequency and dietary diversity practices [33, 34].

It has been well concluded that any interventions that occur after the first 2 years of a child’s life have no significant impact on the growth and development of children [35]. Improving the quantity and quality of a child’s food in this critical windows period is among the most cost effective strategies to improve overall health and ensure nutritional wellbeing [36]. There are strong evidences that appropriate meal frequency and dietary diversity practices lead to better health and growth outcomes among children [37–40]. The role of evidence based health and nutrition information as predictors of minimum meal frequency and dietary diversity practices is significant in improving the levels of inadequate IYCF practices [36], and reducing childhood malnutrition [41]. However, studies are scarce in the predominantly agrarian society of Bale zone, Southeast Ethiopia. With this background in mind, the present study was carried out to identify the determinants of minimum meal frequency and dietary diversity practices among children aged 6–23 months in the zone. The findings the study meant to provide evidences to programme managers and policymakers to design and implement appropriate interventions to improve the levels of inadequate meal frequency and poor dietary diversity practices and reduce childhood malnutrition, morbidity and mortality in the predominantly agrarian society of Bale zone, Southeast Ethiopia.

## Methods

### Study design, time and settings

Community based cross sectional study was conducted from January–June 2016 at Bale Zone, Southeast Ethiopia. Bale zone is one of the 18 zones found in Oromia regional state with a catchment area of 43,690.56 km^2^. According to the 2015 population projection, there are a total of 1,807,279 populations with 376,516 households living in the study area. Bale zone, have a total of 20 woreda and 389 (43 urban and 345 rural) kebeles (the smallest administrative units in Ethiopia) [42]. Furthermore, according to the 2016 Bale Zone Health Department Biannual Health Development report, there are a total of 29, 751 infants and young children aged 6–23 months found in the study area [43].

### Source population, sample size calculation and sampling procedure

The source population was children aged 6–23 months, living in Bale zone, Southeast Ethiopia. Sample size was determined by using a single population proportion formula. The prevalence (P) of minimum meal frequency and minimum dietary diversity practices was taken as 44.7% and 10.8%, respectively [15], with the assumptions of a 95% of confidence level and 5% margin of error (d) for both study factors. Then, the result for minimum meal frequency and minimum dietary diversity scores were 380 and 148, respectively. However, to get a larger value, we took 380 as our required sample size estimation. Finally, a sample size of 836 was obtained after adjusting 2 as a design effect and anticipating 10% as a non-response rate. A multi-stage sampling technique was used in the study. Initially, out of 20 ‘woredas’, four were selected as representative by the lottery method. Kebeles found in the selected woredas were further stratified into urban and rural. A total of 5279 infants and young children aged 6–23 months living in the selected kebeles were obtained from the kebele health post log books and used to calculate the sampling fraction (k). Furthermore, proportional allocation was considered to estimate the total number of children to participate in each selected kebele. Then, a systematic sampling technique was employed to recruit the study participants. The younger child was purposively selected in the households with more than one eligible study subject. When mother-child pairs were not available at the time of data collection, two repeated visits were made.

### Data collection instrument and procedures

An interviewer administered, pretested, and structured questionnaire was used to collect data. The questionnaire was designed with three major sections in mind. The first part was on socio-demographic and economic related characteristics of the child and family. The second part involved dietary practice, co-morbidity status, and health care utilization related characteristics of the child while the third section focused on household environmental and maternal health care utilization characteristics of the household/family. To maintain consistency, the questionnaire was first translated from English to Afaan–Oromo, the native language of the study area, and then retranslated to English by professional translators. A total of 14 health professionals (two experienced public health experts as supervisors and 12 clinical nurses as data collectors) were recruited for the data collection.

The minimum dietary diversity score (DDS) assessment were done by using a 24-h recall method, and the score is used to reflect the micronutrient adequacy of the diet of the infant and young child [44]. DDS were validated for several age/sex groups as proxy measures for both macro and micronutrient sufficiency of the diet [45–47]. Additionally, DDS was significantly associated with the micronutrient density of complementary foods for infants and young children [48]. The tool uses an open recall method to gather information for all food groups and drinks consumed over a given reference period of 24 h. Mothers of infants and young children were requested to list out food groups and drinks consumed by their children in the previous 24 h of the survey. The minimum dietary diversity score was computed based on 7 food groups which contains grains, roots and tubers; legumes and nuts; dairy products; flesh foods; eggs; vitamin–A rich fruits and vegetables; other fruits and vegetables. Likewise, the minimum meal frequency practice of infants and young children was estimated by using a 24-h recall technique. Mothers of children were requested to count their children’s meal frequency in the past 24 h of the interview [49].

Wealth index was used to indicate the socio-economic status of study participants’ households. It was computed by using composite asset indicators for urban and rural residents. A total of 11 composite asset indicators (owning farm land, per-hectare productivity of the farm (in quintals), television set, refrigerator, mobile telephone, availability of electric power, fixed phone, bicycle, cart, number of milk caws and number of oxen) via a principal components analysis (PCA) were used to construct the wealth indexes of households. Finally, the wealth index was classified into five categories (Lowest, Second, Middle, Fourth and Highest) [15].

### Inclusion and exclusion criteria

All infants and young children aged 6–23 months with their mothers and who lives for at least 6 months in the study area were included in the study. However, those infants and young children whose mothers were seriously ill and not able to respond the interviews were excluded from the study.

### Operational definitions

#### Minimum meal frequency

Minimum is defined as proportion of children aged 6–23 months; who receive solid, semi-solid, or soft foods at the minimum numbers of two and three times for children aged 6–8 months, and 9–23 months respectively [49].

#### Minimum dietary diversity score

Is proportion of infants and young children aged 6–23 months who received foods and drinks from ≥4 food groups in the previous 24 h [49].

#### Afaan-Oromo

Is an Afro-asiatic and Cushitic family’s language spoken by about 30 million people in Ethiopia [50].

#### Woreda

Is the third level administrative divisions in Ethiopia. It contained an estimated population size of 50,000–150,000 in its catchment [51].

#### Kebele

Is the smallest administrative unit in Ethiopia. It contained an estimated population size of 5000–15,000 in its catchment [51].

### Data quality issues

A training of 3 days was given to recruited data collectors and supervisors. The training mainly focused on equipping the trainees with information about the objective of the study, techniques of interview, collection of data, and relevant ethical issues. The data collection tool was pretested on 5% of the study subjects out of the selected kebeles. During the pre–test, the acceptability and applicability of the procedures and tools were evaluated. All questioners were regularly checked for completeness, clarity, and consistency by the respective supervisors. The investigators coordinated the overall activities of data collection. Data validity and reliability were maintained through a close supervision of data collectors and supervisors by the investigators.

### Data processing and analysis

Data was entered into EPI INFO version 3.5.3 and analyzed by using Statistical Package for Social Sciences (SPSS) version 20. Descriptive statistics, including frequencies and proportions were used to summarize the study variables. A binary logistic regression model was used to find out factors associated with minimum meal frequency and minimum dietary diversity practices. Those variables in both minimum meal frequency and dietary diversity score a P–Values of <0.2 in the bivariate analysis were transferred to the multivariable analysis to control the possible effect of confounders. The Adjusted Odds Ratio (AOR) with a 95% of confidence intervals was used to notify the strength of association, and at P–Values of ≤0.05 was used to declare the statistical significance in the multivariable analysis.

## Results

### Socio–demographic and economic characteristics related variables of the child and the family

A total of 801 mother-child pairs with a response rate of 95.8% were included in the final data analysis process. The mean age of mothers/caretakers was 26.5 years with nearly half of them in the age ranges of 25–35 years. About 16.9, 17.4, and 65.8% of infants and young children were aged 6–8, 9–11, and 12–23 months respectively. More than 70 % of the mothers of the infants and young children attended formal education. About 18.9 and 19.7% of households were found at the lowest and highest socio-economic levels, respectively (Table 1).

**Table 1:** Socio–demographic and economic related characteristics of children aged 6–23 months and the family at Bale zone, Southeast Ethiopia, 2016(*n* = 801)

Characteristics	Category	Frequency	Percentage
Age of the child (in months)	6–8 month	135	16.9
	9–11 months	139	17.4
	12–23 months	527	65.8
Sex of the child	Male	468	58.4
	Female	333	41.6
Birth order of the child	1st	245	30.6
	2nd	199	24.8
	3rd	125	15.6
	≥ 4th	232	29.0
Maternal age (in years)	< 25 years	312	39.0
	25–35 years	393	49.0
	≥ 36 years	96	12.0
Religion	Muslim	503	62.8
	Orthodox	258	32.2
	Protestant	34	4.2
	Catholic	6	0.7
Maternal marital status	Married	752	93.9
	Single	23	2.9
	Divorced	26	3.2
Mothers educational level	Illiterate	195	24.3
	Informal education	31	3.9
	Formal education	575	71.7
Employment status of the mother	House wife	612	76.4
	Farmer	49	6.1
	Governmental employed	50	6.2
	Daily labourer	10	1.2
	Merchant	80	10.0
Educational status of the father	Illiterate	119	15.8
	Informal education	44	5.9
	Formal education	589	78.3
Employment status of the father	Farmer	416	55.3
	Merchant	166	22.1
	Governmental employed	125	16.6
	Daily labourer	45	6.0
Household family size	≤ 3	246	30.7
	4–6	416	51.9
	≥ 7	139	17.4
Household wealth index	Lowest	151	18.9
	Second	170	21.2
	Middle	160	20.0
	Fourth	162	20.2
	Highest	158	19.7
Maternal exposure to different medias	Yes	499	62.3
	No	302	37.7

### Dietary practice and co-morbidity status related characteristics of children

More than 91 percents of study subjects were breast feeding during the interview. About 73.5% of study subjects were participated growth monitoring in the previous 1 month of the survey. As mothers of children confirmed that, more than 20 % of children were experienced complaints of illness in the past 1 week of the survey (Table 2).

**Table 2:** Dietary practice and co-morbidity status related characteristics of children aged 6–23 months at Bale zone, Southeast Ethiopia, 2016 (*n* = 801)

Characteristics	Category	Frequency	Percentage
Currently breast feeding	Yes	734	91.6
	No	67	8.4
Growth monitoring in the in past one month	Yes	589	73.5
	No	212	26.5
Child history of illness in the past 1 week	Yes	179	22.3
	No	622	77.7
Timely initiation of complementary foods	Yes	498	62.2
	No	303	37.8
Minimum meal frequency	Yes	548	68.4
	No	253	31.6
Minimal dietary diversity scores	Yes	228	28.5
	No	573	71.5
Minimal acceptable diet	Yes	207	26.8
	No	594	73.2

### Food groups consumed by children aged 6–23 months in the previous 24 h of the survey

**Fig. 1:**
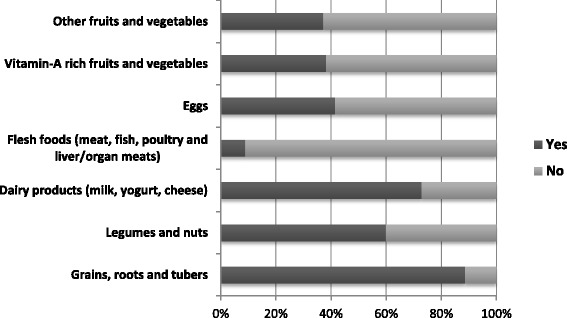
Types of food groups consumed by children aged 6–23 months in the previous 24 h of the survey at Bale zone, southeast Ethiopia, 2016

Proportion of infants and young children aged 6–23 months, who practiced a minimum dietary diversity scores were28.5% [95% CI: 0.254, 0.316]. More than 19 % of infants and young children was received foods prepared from grains, roots and tubers. Only 8.9% of children were consumed flesh foods in the previous 24 h of the interview (Fig. 1).

### Household environmental and maternal health care utilization characteristics

More than 75 % of children mothers had ≥ 4 times of antenatal care (ANC) service visit to the nearest health institution during their child pregnancy life. While, about 68.5% of mothers had ≥ 3 times postnatal care (PNC) service visits during the interview. Beside to this, near to 60 % of mothers was received nutrition counselling on IYCF practices by health professionals during their visit. Latrine was available in more than 95 % of study participants household (Table 3).

**Table 3:** Household environmental and maternal health care utilization characteristics of children aged 6–23 months at Southeast Ethiopia, 2016 (*n* = 801)

Characteristics	Category	Frequency	Percentage
Parity of the mother	1–2	432	52.8
	3–6	321	40.1
	≥ 5	57	7.1
History of antenatal care visit	Yes	742	92.6
	No	59	7.4
Frequency of antenatal care visit	1–3	169	22.8
	≥ 4	1573	77.2
Counselling on infant and young child feeding during antenatal care visit visit	Yes	445	60.0
	No	297	40.0
Postnatal care service visit	Yes	676	84.4
	No	125	15.6
Frequency of postnatal care service visit	1 Times	42	6.2
	2 Times	171	25.3
	≥ 3 times	463	68.5
Counselling on infant and young child feeding during postnatal visit	Yes	402	50.2
	No	274	34.2
Household source of drinking water	Protected	691	86.3
	Unprotected	110	13.7
Availability of home garden	Yes	256	32.0
	No	545	68.0
Availability of latrine	Yes	763	95.3
	No	38	4.7

### Factors associated with minimal meal frequency

The bivariate logistic regression analysis showed that child age, household family size, parity of mother, maternal counselling status on IYCF during ANC and PNC service visits were associated with minimal meal frequency. However, in the multivariate regression analysis only the age of the child and parity of the mother remained to affect minimum meal frequency practice among children aged 6–23 months. As a result, the odds of minimum meal frequency were 71% times less likely among children aged 6–8 months than those aged 12–23 months (AOR = 0.29, 95% CI: 0.28–0.94). Parity of the mother was another factor significantly associated with minimum meal frequency. As a result, para-one and two mothers were 2.8 times more likely to practice minimum meal frequency to the child than para-five and above mothers (AOR = 2.8, 95% CI: 1.11–7.50) (Table 4).

**Table 4:** Bivariate and multivariate analysis of factors associated with minimal meal frequency among children aged 06–23 months at Bale zone, Southeast Ethiopia, 2016

Characteristics	Minimal meal frequency				
	Category	Yes	No	COR (95% CI)	AOR (95% CI)
Household family size	≤ 3	169	77	1.6 (1.05,2.49)	0.9 (0.42,1.95)
	4–6	299	117	1.9 (1.26,2.80)	1.48 (0.81,2.7)
	≥ 7	80	59	*1.00*	*1.00*
Parity of the mother	1–2	308	115	2.5 (1.47,4.53)	2.8 (1.11,7.50)*
	3–4	211	110	1.8 (1.04,3.26)	1.5 (0.69,3.66)
	≥ 5	29	28	*1.00*	*1.00*
Age of the child	6–8	67	68	0.26 (0.18,0.40)	0.29 (0.28,0.94)*
	9–11	67	72	0.25 (0.17,0.37)	0.24 (0.15,0.39)*
	12–23	414	113	*1.00*	*1.00*
Counselling on IYCF during ANC visit	Yes	328	117	1.83 (1.33,2.49)	1.28 (0.79,2.05)
	No	180	117	*1.00*	*1.00*
Counselling on IYCF during PNC visit	Yes	302	100	1.93 (1.38, 2.69)	1.25 (0.78,1.99)
	No	167	107	*1.00*	*1.00*

***Note*** : *Significant at *P*-Value <0.05; DDSs–Dietary Diversity Scores; COR–Crude Odds Ratio; AOR–Adjusted Odds Ratio

### Factors associated with minimal dietary diversity scores (DDSs)

Maternal educational status, household family size, level of exposure to different media, parity of mother, child illness in the past 1 week of the survey, mothers counselling status on IYCF during ANC, and PNC service visits were identified as factors associated with minimum dietary diversity score in the bivariate regression analysis. However, in the multivariate analysis, mothers’ educational status, child illness in the past 1 week of the survey, and maternal counselling status on IYCF during PNC service visits were factors statistically associated with minimal dietary diversity practice in the study area. Accordingly, minimum dietary diversity practice of study subjects was 48% times less likely among illiterate mothers than mothers with formal education [AOR = 0.52, 95% CI: 0.28–0.94]. A child’s history of illness in the week preceding the interview decreased the practice of minimum dietary diversity scores by 56% times [AOR = 0.44, 95% CI: 0.26–0.73]. Likewise, the odds of minimum dietary diversity practice of the child were 2.6 times higher among mothers counselled on IYCF practice during PNC visits [AOR = 2.6, 95% CI: 1.59–4.51] (Table 5).

**Table 5:** Bivariate and multivariate regression analysis of factors significant with minimal DDSs among children aged 06–23 months at Bale zone, Southeast Ethiopia, 2016

Characteristics	Minimal dietary diversity practice				
	Category	Yes	No	COR (95% CI)	AOR(95% CI)
Educational status of the mother	Illiterate	36	159	0.46 (0.32,0.69)	0.52 (0.28,0.94)*
	Informal education	4	27	0.30 (0.10,0.88)	0.20 (0.04,0.91)*
	Formal education	188	387	*1.00*	*1.00*
Household family size	< 4	80	166	1.9(1.16–3.12)	2(0.89–4.5)
	4–6	120	296	1.6 (1.0,2.56)	1.2 (0.60,2.37)
	>6	28	11	*1.00*	*1.00*
Exposure to different media	Yes	167	322	1.96 (1.41,2.78)	1.27 (0.82,1.95)
	No	61	241	*1.00*	*1.00*
Parity of the mother	1–2	129	294	3.13 (1.38,7.0)	1.6 (0.48,5.53)
	3–6	92	229	2.8 (1.25,6.56)	2.5 (0.84,7.88)
	≥ 7	7	50	*1.00*	*1.00*
Child illness in the past 1 week	Yes	34	145	0.51 (0.34,0.78)	0.44 (0.26,0.73)*
	No	194	428	*1.00*	*1.00*
Counselling on infant and young child feeding during antenatal care visit	YES	162	283	2.4 (1.7,3.4)	1.57 (0.92,2.6)
	No	57	240	*1.00*	*1.00*
Counselling on infant and young child feeding during postnatal care visit	Yes	149	253	4.1 (2.7,6.2)	2.6 (1.59,4.45)*
	No	34	240	*1.00*	*1.00*

***Note*** : *Significant at *P*-Value <0.05; DDSs–Dietary Diversity Scores; COR–Crude Odds Ratio; AOR–Adjusted Odds Ratio

## Discussion

The proportion of children aged 6–23 months who received the recommended minimal meal frequency and minimum diversified food groups was 68.4% [95% CI: 0.652, 0.716] and 28.5% [95% CI: 0.254, 0.316], respectively. In the present study, the minimum meal frequency was higher than reports from Ethiopia [44.7 and 50.4%] [15, 21], Ghana [57.3%] [52], Democratic Republic of Congo (30%] [53], and India [42%] [31]. The possible reasons for the dissimilarity between studies could be the difference in religious and socio-cultural beliefs, all of which can influence appropriate infant and young child feeding practices [16]. The role of regional differences among studies highlights the importance of ensuring interventions to improve proper child feeding practices in the local contexts [13]. Another possible explanation may be the time gap among studies, i.e. the previous local study [15] was carried out over 5 years ago. However, our finding on minimum meal frequency was lower than the [82.0%] of reported in Ethiopia [22] and [71.1%] [30], [81%] [54], and [84%] [55] in such Asiatic and Latin American nations, like Sri Lanka, Bangladesh, and Nepal, respectively. The possible reason for the variability among reports could be the difference in socio–economic status of the household, which can further show the purchasing power of families to feed their children. In this study, more than 60 % of children’s households were found in the middle and low level of wealth status. The important role of household resource is that it helps in determining the quality and quantity of complementary feeding for the child [33]. Furthermore, it may be due to variations in the degree of health seeking behavior and knowledge on IYCF practices among study communities. The present finding for minimum dietary diversity practice was somewhat better compared to previously documented results in Ethiopia [10.8–12.6%] [15, 21], Burkina Faso [14%] [56], Mali [16%] [48], and India [15.2%] [31]. On the other hand, this finding on minimum dietary diversity practice was lower than that of studies done in Ethiopia [39.1%] [22], and other developing nations, like Nepal [34%] [57], India [33%] [58], Bangladesh [41.9%] [54], and Sri Lanka [71%] [30]. The possible reason for the differences among studies might be due to variations in study settings, sample size consideration, and time gap among studies. Our study was conducted on a small sample size. But, in previous local study [15], more heterogeneous and larger study community was participated in different settings with varying socio-cultural beliefs on infant and young child feeding practices.

Child age was significantly associated with minimum meal frequency. The odds of minimum meal frequency were lesser among children aged 6–11 months than children aged 12–23 months. This indicates that minimum meal frequency is positively associated with the age of infants and young children, implying that the practice of minimum meal frequency increases as the age of children increases. The result was in line with those of studies conducted in Ethiopia [21], Ghana [52], and other Asian countries, like India [58] and Sir Lanka [30], i.e., children aged 6–11 months independently had higher risks for decreased minimal meal frequency practice than their counter parts. This was further evidenced by the fact that, age is an important consideration in assessing meal frequency and dietary adequacy of infant and young child feeding practices [21]. This is again testified by the fact that as the child grows older the frequency of meal increases and diets become more diverse. Additionally, it is because eruption of the teeth influences when the child is introduced to complementary foods, but the process typically leads to loss of appetite and negatively affecting the frequencies of meals for the child [29, 59]. Besides, it may be due to the maternal perception of the younger child and the low ability of the child’s intestine to digest and absorb foods. Moreover, mothers may perceive that introducing children to bulks of food would lead to developing many types of infections [60]. Furthermore, these findings show the importance of considering the two events, i.e., practicing age appropriate meal frequency and risks of childhood malnutrition. In most developing countries, infant and young child malnutrition due to inappropriate feeding occurs in the first 2 year of the child’s life [13]. The lives of many infants and young children can be saved through a range of cost-effective actions and increasing the rates of age appropriate meal frequency and other IYCF practice indicators at large. Interventions targeted at identifying practices, beliefs, concerns, and constraints relating to minimum meal frequency and addressing them through appropriate messages and counseling have a key role in the health and nutritional status of infants and young children [61].

The number of child births of the mother was another possible factor for attaining minimal meal frequency. The odds of minimal meal frequency increase nearly three times among children of para ≤ two mother. The possible reason may be that a mother with fewer childbirths may have a better commitment and good motivation to feed and care her child appropriately. Additionally, having fewer childbirths may increase mothers’ level of love for children which may inversely influence the level and type of child care and feeding practices. Another possible explanation could be that mothers with few childbirths may have better level of education than multi–para mothers. This is due to the fact that mothers’ level of education can strongly influence on the decision of having number of childbirths in their reproductive age [62]. As well, educated mothers are more inclined to practice scientifically proven feeding recommendations for their children [63].

Unlike other studies [22, 53, 54, 58], no factor was identified to have a significant association with both minimum meal frequency and dietary diversity practices in this study. After we controlled the possible effects of other factors in the multivariate regression analysis mothers’ education, child illness in the past 1 week of the survey and maternal counseling on IYCF practices during PNC service visits remained determinant factors for minimum dietary diversity practice in this study community.

Maternal educational status was significantly associated with minimum dietary diversity practice of infants and young children. Mothers with better educational status were more likely to practice minimum dietary diversity for their children. The importance of mothers’ education for child health, degree of nutrition and feeding practice has been well investigated in Ethiopia [64], Nepal [55], Bangladesh [54] and India [58]. This is highly supported by the fact that, mother’s education has a strong role in increasing thier level of confidence, command over resources independently, and adherence to recommended IYCF practices [64, 65]. Education is a proxy measure for socioeconomic wellbeing and researchers argue that mothers’ with better education tend to have more work opportunities and better wealth than mothers’ with lower educational status [60, 66]. This could increase their purchasing power of different food groups and feeding diversified foods for their children.

Child illness in the week preceding the survey influenced minimum dietary diversity practice of infants and young children. The present finding was comparable with those of studies done in Ghana [52] and India [67], indicating that child illness in the week absolutely affected the dietary diversity practice of children. This could be due to the fact that common childhood illnesses are known to affect the type of food and feeding practice by reducing the child’s appetite [59]. In addition, due to the illness of the child, mothers would reduce giving large amount and different types of food groups to the children.

Maternal PNC service attendance at health institutions was the other possible risk factor for minimum dietary diversity practice in the study area. The risk of not practicing minimum dietary diversity for the child was higher among mothers who had no PNC service visits. The result was in congruence with those of studies in Ethiopia [21] and other developing nations, like Sri Lanka [30], India [67] and Tanzania [68]. These studies showed that mothers’ lack of heath institution visits during PNC periods positively determine the dietary diversity practice of infants and young children. This might be due to the fact that mothers’ health institution visits during the PNC period has potential opportunities for their getting health and nutrition knowledge by trained health workers on IYCF practices [69]. Health workers have a crucial role in supporting mothers by providing nutrition counseling about balanced diets and appropriate IYCF practices [70]. Thus, they help in ensuring good nutrition in the first couple years of the child’s life [34]. Furthermore, this counseling may polish mothers’ cultural and traditional beliefs and bring positive influences on IYCF practices [71]. Mothers counselled on IYCF during PNC visits may become more informed about the importance of providing diversified food groups to their children. Thus, intensified efforts are needed to improve the level of PNC service utilization and to provide nutrition counseling to mothers on IYCF practices.

To sum up, some limitations of the study have to be noted. First, as the design of the study was cross-sectional, it was difficult to examine potential temporal relationships. Second, co–morbidity status of the child was conveyed only in the information given by mothers’. This might be subjected to bias as it depends on the mothers’ level of knowledge about the illness status of the child. Third, the measurement of child feeding practice again relied on memory, so there may be a possibility of recall bias. Its being unrepresentative of the whole nation is another possible limitation of the study. Nevertheless, the study has successfully showed important trends that can be used in the formulation of other studies and interventions to improve the IYCF practice in the study area.

## Conclusion

This study was found low minimal meal frequency and dietary diversity practice in the predominantly agrarian society of Bale zone, Southeast Ethiopia. No factors were identified to have statistical association with both minimal meal frequency and dietary diversity practice. Child age and parity of mother was the only study variable to have significant association with minimal meal frequency. However, educational status of mothers, child illness in the preceding week of the interview and maternal counselling on IYCF during PNC service visits were independently influenced minimum dietary diversity practice. Improving the level of maternal and child health care utilization, increasing mothers’ level of education, and providing health, and nutrition counseling on IYCF during maternal PNC service visits are vital interventions to improve IYCF practices in the study area.

## Abbreviations

ANC: Ante Natal Care
AOR: Adjusted Odds Ratio
CI: Confidence Interval
COR: Crude Odds Ratio
DDS: Dietary Diversity Score
IYCF: Infant and Young Child Feeding
PNC: Post Natal Care
SPSS: Statistical Package for Social Science
WHO: World Health Organization
